# Prosthetic management of an epulis fissuratum with simple conditioning tissue: A case report

**DOI:** 10.1016/j.ijscr.2024.110130

**Published:** 2024-08-08

**Authors:** El Ghmari widad, Merzouk nadia, Slaoui Hasnaoui jihane

**Affiliations:** a Resident dentist in the Removable Prosthetics Department; Faculty of Dental Medicine of Rabat, Mohammed V University in Rabat, Morocco; b Professor and head of the Removable Prosthetics Department, Faculty of Dental Medicine of Rabat, Mohammed V University in Rabat, Morocco; c Professor in the Removable Prosthetics Department, Faculty of Dental Medicine of Rabat, Mohammed V University in Rabat, Morocco

**Keywords:** Epulis fissuratum, Conditioning tissue, Ill-fitting denture

## Abstract

**Introduction and importance:**

Epulis fissuratum is an oral hyperplasic lesion related to an overextended and ill-fitting denture. The treatment of this lesion can be conservative or surgical associated to a good prosthetic management.

**Case presentation:**

The aim of this article is to present a case report about treatment of an epulis fissuratum with simple conditioning tissue, using provisional removable complete dentures.

**Clinical discussion:**

The early management of the epulis fissuratum with conditioning tissue, associated to a good oral hygiene and a correct prosthetic rehabilitation provides satisfactory clinical results and avoids surgical treatment.

**Conclusion:**

Conditioning tissue technique is a useful conservative approach for the treatment of the epulis fissuratum at an early stage.

## Highlights

• This case report presents a conservative approach for treatment of an epulis fissuratum with simple conditioning tissue, using provisional removable complete dentures.
• This technique is interesting especially if surgical treatment is contraindicated, or if the patient refuses any surgical intervention to remove his hyperplasic lesion, which is the case for our patient.
• The early management of this denture-related oral lesion is interested to avoid any invasive surgical treatment.
• This technique should be associated with a good oral and denture hygiene.

## 1 Introduction

The epulis fissuratum is an oral hyperplasic lesion associated to wearing of ill-fitting dentures. It refers to a reactive tissue response to excessive mechanical pressure imparted by the poor fit of denture. It is defined as an inflammatory fibrous hyperplasia or denture-induced fibrous hyperplasia (1). It is most found in the mucolabial and mucobuccal folds of the mandible or in the maxilla and arise secondary to irritation from a denture flanges. It is a result of the constant trauma and inflammation caused by the pressure from overextended denture borders or tipping forces resulting from an imbalanced occlusion.

The diagnosis of this lesion is clinical, epulis fissuratum is described as a raised, sessile with single or multiple folds. Most often there are two folds of tissue, one extending beneath the denture and one extending over the polished surface of the flange. Although often covered by an intact mucosa, the lesions may sometimes be ulcerated (2,3). Histologically, the lesions are composed of an increased quantity of fibrous tissue with varying numbers of chronic inflammatory cells, which are predominantly plasma cells. The differential diagnosis of fibrous inflammatory hyperplasia should include consideration of the possibility that the lesion is a true papilloma, or a squamous cell carcinoma that has proliferated around a denture flange (3,4).

The treatment of this oral lesion could be conservative or surgical. The surgical treatment is managed by excision and biopsy to eradicate the lesion and to rule out a possible malignancy and by relining or remaking the prosthetic appliance that was presumably the inciting agent (5). The surgical excision of the epulis fissuratum causes bleeding, post operative pain and edema. The conventional surgical procedure requires sutures and sometimes tissue grafts, consequently, loss of the depth of the vestibule, delayed wound healing and re-epithelialization. Recurrence may be a problem following excision if the ill-fitting denture is not repaired or replaced (5,6). This is why non-surgical treatment, especially if the epulis is small and diagnosed earlier, is a conservative approach that avoids the post-operative consequences of surgical treatment.

Studies in the literature agree that epulis fissuratum is most often located in the anterior region of both maxillae. In the study by Buchner et al. (2), 55 % of lesions were in the maxilla and 45 % in the mandible. The age of predilection at the time of discovery or surgical excision is the sixth and seventh decades with a female predomination. It has no malignant potential, and recurrences following excision are almost always a result of the failure of elimination of chronic irritation involved by sharp and overextended prosthetic edges (4).

We presented a case report of a patient presenting an epulis fissuratum associated to an overextended and ill-fitting removable complete denture. He has been received in the removable prosthodontics service and treated with soft conditioning tissue technique to remove his oral lesion. The aim of this case report is to demonstrate the usefulness of this conservative therapeutic in the treatment of this oral lesion. This work has been reported in line with the SCARE criteria (7).

## 2 Patient information

A 66 years-old male patient was referred to the Department of Removable Prosthodontics for prosthetic rehabilitation. The medical history revealed uncontrolled type 2 diabetes and arterial hypertension treated with beta blockers. The patient has no smoking history. He reported denture instability during mastication and phonation, and nocturnal wearing of his prosthesis. The patient does not brush his dentures for long years, and he reported any oral or prosthetic hygiene methods.

## 3 Clinical findings

An exobuccal examination revealed a diminution of vertical dimension of occlusion and a laterodeviation. The endobuccal examination revealed bimaxillary complete edentulous supporting complete dentures, poor oral hygiene, and a generalized palatal erythema. The lower arch revealed an inflammatory hyperplasia localized in the mandibular vestibular sulcus and appeared 3 months ago. The oral lesion was movable and non-hemorrhagic during palpation. No cervical lymphadenopathy was evident. The prosthetic examination revealed an ill-fitting and unproper removable complete dentures, an overextended prosthetic in contact with the mandibular hyperplasia with thin and sharp edges. The occlusal examination of the poor prostheses showed inverted articulated malocclusion and poorly distribution of general occlusal contacts.

### 3.1 Radiological examination

Panoramic radiograph revealed moderate resorption level of bimaxillary edentulous ridges.

## 4 Timeline

After the first visit, the patient was referred with a letter to his doctor to ascertain the stability of his hypertension and the presence of any concomitant general illnesses. 1 week later, the patient was seen again. His doctor's response revealed that his hypertension was stable. The patient began anti-diabetic treatment to balance his diabetes, then prosthetic treatment can be started. The patient is advised not to wear their old prostheses during this period until refection of new provisional prostheses.

## 5 Diagnostic assessment

**Fig. 1:**
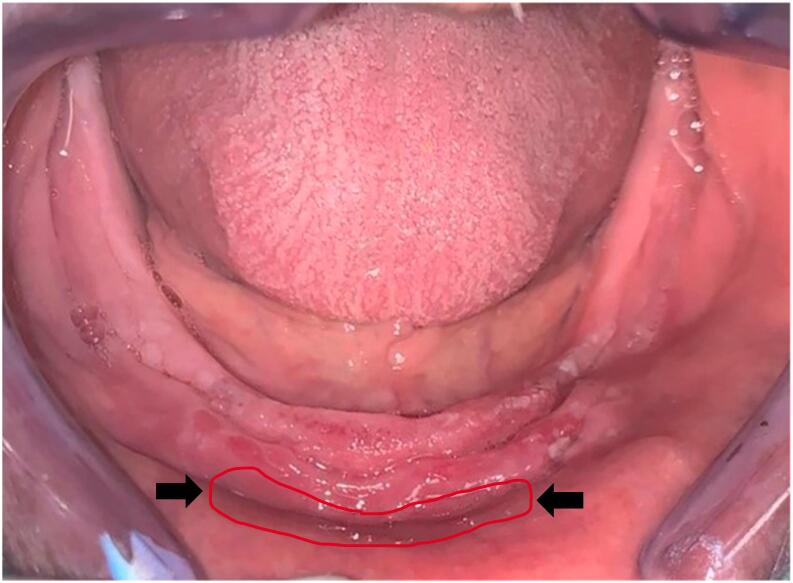
Preprosthetic management of the epulis fissuratum in the mandible.

Basing on clinical and radiologic findings, the diagnosis was a denture stomatitis Newton type II in the maxillary arch, and an epulis fissuratum in the lower arch related to the overextended and ill-fitting denture base and lack of denture maintenance **(**Fig. 1**).**

## 6 Therapeutic intervention

The conservative therapeutic was the treatment of choice for these dentures oral lesions because the patient has refused any surgical intervention of his epulis fissuratum. After refection of new temporary removable complete dentures, the tissue conditioning treatment was carried out using a tissue conditioner, the material has been renewed until the regression of the lesion. The patient has been informed about methods of the oral and prosthetic hygiene.

### 6.1 Prosthetic management

#### 6.1.1 Refection of provisional removable complete dentures

After primary and secondary impressions, the patient's maxillomandibular relationship has been taken using wax rims. After selection of resin artificial teeth, denture teeth arrangement has been made. Esthetic and functional fitting is carried out to have patient's agreement. Then provisional complete prostheses have been inserted after polymerization of dentures in the laboratory. Occlusal equilibration has been made for best prosthetic integration.

#### 6.1.2 Tissue conditioning

**Fig. 2:**
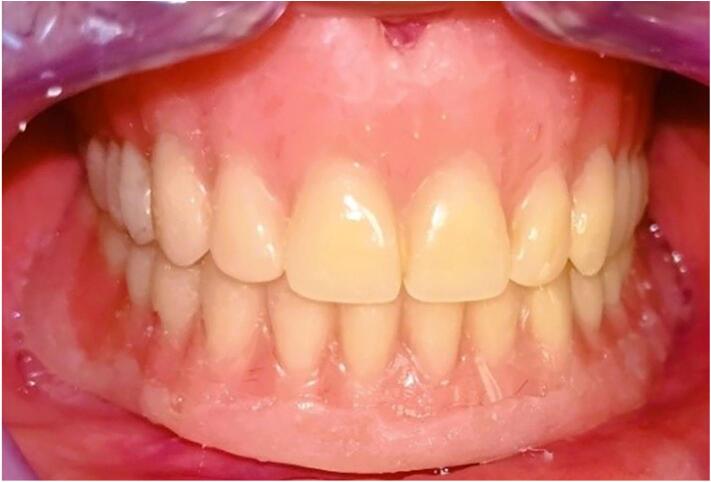
Conditioning tissue using provisional removable complete dentures.

The provisional complete dentures have been used for tissue conditioning using a tissue conditioner (*GC Soft Liner®)* for the treatment of both oral lesions, maxillary denture stomatitis and the epulis fissuratum in the mandible. The material has been charged into prosthesis intrados and placed into the patient's mouth. The patient was then asked to perform allfunctional movements. The initial setting of the tissue conditioner was carried out under prosthetic occlusal pressure **(**Fig. 2**)**. The prosthetic borders related to the epulis fissuratum were smoothened by adding and molding the tissue conditioning material so that the prosthetic borders will progressively print a soft and continuous tissue pressure to the hyperplasic lesion. The prosthetic edges were thick and rounded. The tissue conditioning material was renewed every week for 2 months. This operation was repeated until a total regression of the epulis fissuratum. A medical prescription was associated to the prosthetic management for treatment of denture stomatitis using local antifungal treatment (Daktarin®) for 3 weeks. Dentures oral hygiene instructions were provided, the patient was advised to brush his oral mucosa and prostheses daily and not to use the dentures at night. A daily massage of his mandibular hyperplasia for 10 minutes is recommended for best regression of the oral lesion.

## 7 Follow up and outcomes

**Fig. 3:**
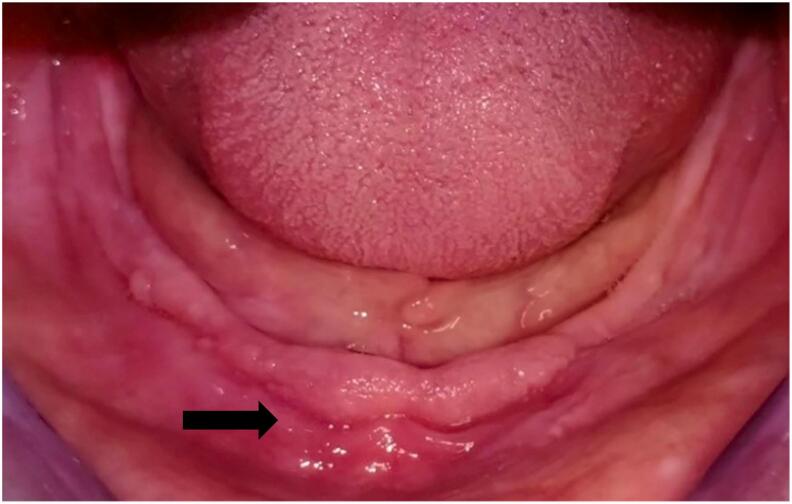
Total regression of the epulis fissuratum after 2 months of tissue conditioning treatment.

The patient was seen after 48 h to control his dentures adaptation. Occlusal equilibration was made to remove any traumatic factor. After 2 weeks, the palatal inflammation was regressed, and the size of mandibular hyperplasia was reduced. For 2 months, the soft conditioner has been renewed every week after total regression of the epulis fissuratum **(**Fig. 3). The provisional removable complete dentures were used for confection of the definitive removable complete dentures. A low-viscosity silicone surfacing impression is made under occlusal pressure. The maxillary arch transfer is made using facebow. The mandibular arch transfer is made using TENCH articulated technique, in centric relation and maintaining the vertical occlusal dimension. The choice of prosthetic teeth was made according to the classic rules. Then, an esthetic and functional fitting is carried out. After polymerization, prosthetic insertion and occlusal equilibration are performed. Oral hygiene and prosthetic instructions are then given to the patient. An annual check-up is prescribed to the patient to detect an eventual recurrence of his oral lesions. The patient was satisfied with his new dentures.

## 8 Discussion

Denture related oral lesions are a heterogeneous group of tissue changes associated to the wearing of ill-fitting dentures. These lesions are found to be more frequent in female than in male ([8], [9], [10]). Several etiological factors are associated to oral lesions. Acute and chronic irritation from ill-fitting dentures may injure the oral mucosa. Other risk factors such as age, quality and quantity of saliva produced, medical conditions, smoking, chewing tobacco, nocturnal denture wearing, oral and prosthetic hygiene may affect the integrity of oral mucosa and then the occurrence of oral lesions (11).

The aim of this case report is to show usefulness and efficiency of tissue conditioning approach in the treatment of the epulis fissuratum, associated to the wearing of ill-fitting dentures.

The treatment of the epulis fissuratum varies according to the severity of the inflammation and the quantity of redundant tissue that is involved. If the condition is incipient, tissue conditioning can be used to treat these lesions without the need of the surgical treatment. This technique also increases the prosthetic bearing surface and biofunctional space, thereby enhancing prosthetic balance and stability, especially in the case of highly resorbed edentulous ridge. If the old denture must be worn for esthetic reasons, the flange can be removed or modified, and the denture is lined with a tissue conditioning material. However, if the condition is chronic with very large, old and fibrous hyperplasia, the epulis should be surgically removed. The offending flange can be modified following surgery and the denture lined with tissue conditioner can then be worn as a surgical splint while healing takes place. A split mucosal graft technique can be effective to deepen the height of the residual alveolar ridge and to increase the amount keratinized tissue if the lesion was very large and extensive. The denture can be refitted or remade when healing is complete (12,13).

The surgical therapeutic with appropriate prosthetic reconstruction had been largely described in literature for the treatment of the epulis fissuratum. Several surgical techniques can be used for its surgical excision using conventional surgery, electric scalpel surgery, cryosurgery, surgery using the carbon dioxide laser, Erbium-YAG laser, Neodymium: YAG laser, or diode laser. The use of laser carbon dioxide (CO2) in the treatment of this lesion presents many advantages over conventional surgery, including less surgery time, less bleeding during surgery, more vestibular depth, better reepithelialization of the wound and less need of suturing which provide satisfactory results in oral function and tissue health, and may be clinically preferred method for surgical treatment of epulis fissuratum (14,15).

In our case report, we had chosen conservative approach, using conditioning tissue in the treatment of the lesion for 2 reasons, because it was detected earlier, and secondly, the patient has refused any surgical intervention. Because of the presence of comorbidities (uncontrolled diabetes melitus type II and arterial hypertension), surgical treatment in this case exposes the patient to an infectious risk, which justifies antibiotic prophylaxis and antibiotic therapy before and after treatment.

A follow-up period of 2 months was sufficient to remove this lesion, the tissue conditioner material has been renewed every week, associated to a good oral and prosthetic hygiene. A long-term follow-up of 2 years were instituted, to control any recurrence of the lesion, combined with an occlusal equilibration of his complete dentures, which further enhanced prosthetic integration.

Persistent trauma to oral mucosa caused by a poorly fitted prosthesis may be a predisposing factor for recurrence of the lesion and development of carcinoma. Hence, the importance of periodic check-ups for patients wearing removable complete dentures, with a short- and long-term regular control schedule, to confirm the diagnosis of the lesion at an early stage, and to rule out any malignant transformation.

### 8.1 Conclusion

The conservative therapeutic with conditioning tissue is a useful approach for treatment of the epulis fissuratum, especially if the lesion is not very extensive and diagnosed earlier which was the case with our patient.

### 8.2 Patient perspective

The patient expressed great satisfaction with the outcome of the conservative management of his epulis fissuratum by tissue conditioning, noting improved prosthetic stability and oral function, particularly during mastication.

## Informed consent

Written informed consent was obtained from the patient for publication of this case report and accompanying images. A copy of the written consent is available for review by the Editor-in- Chief of this journal on request.

## Ethical approval

Our case report is exempt from ethical approval.

## Funding

Not applicable (N/A).

## Author contribution

• El Ghmari widad: Writing the paper.
• Merzouk nadia: Correction of the paper.
• Slaoui Hasnaoui jihane: Correction of the paper.

## Guarantor

- Dr. El Ghmari widad.
- Dr. Slaoui Hasnaoui jihane.

## Declaration of competing interest

The authors declare no conflicts of interest.
